# Supplementary figures and images for: pET28g: A Golden Gate-compatible pET vector for protein expression in Escherichia coli, validated by production of functional human ACE2
Source: PLoS One. 2025 Jul 7;20(7):e0327341. doi: 10.1371/journal.pone.0327341 (PMC12233280; doi:10.1371/journal.pone.0327341)

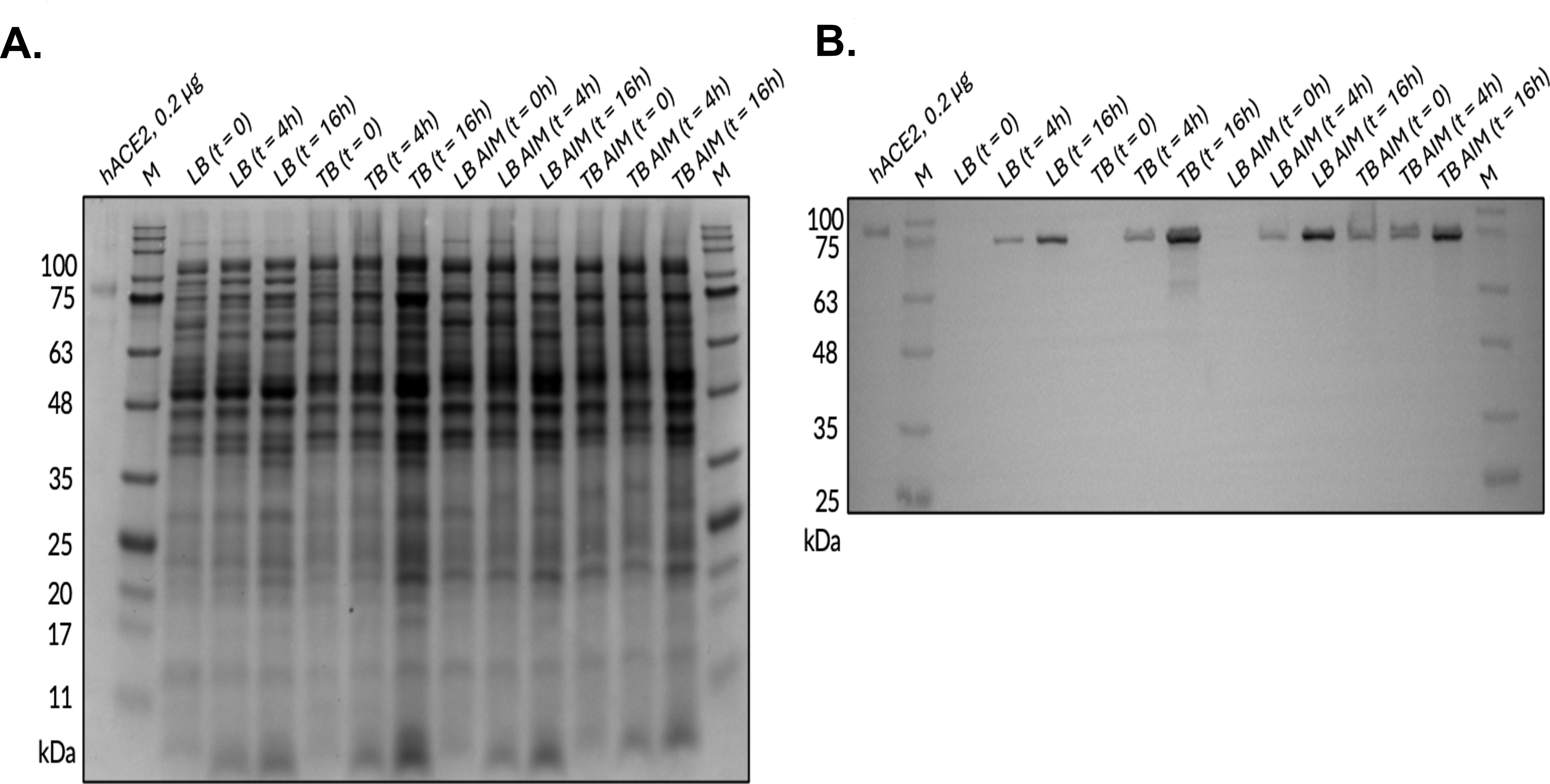

Supplement: S1 Fig — (A) 10% SDS-PAGE analysis of soluble protein fractions, prepared as described in the Materials and Methods subsection ACE2 production and purification using pET28g-Gb1_ACE2. Samples were induced with IPTG and subjected to a temperature shift from 37 °C to 18 °C for cultures grown in LB and TB media, while cultures in LB AIM and TB AIM were induced only by the temperature shift from 37 °C to 18 °C. Protein expression was analyzed at the indicated time points (0, 4 h, 16h). (B) Western blot analysis of the various conditions using a polyhistidine antibody (Sigma-Aldrich, H1029, 1:3000 dilution). A distinct band at approximately 69 kDa confirms ACE2 expression in the soluble fractions. TB AIM with induction at 18 °C for 16 h shows the most intense and well-defined band at ~ 75 kDa, indicating the highest soluble yield of soluble ACE2. The other conditions show weaker signals or higher background. As these analyses focus on soluble fractions, inclusion bodies are not visible on these gels. Based on these results, TB AIM media with induction at 18 °C for 16 h was selected for the for scale-up production of ACE2. M, molecular weight marker. (TIF) [file pone.0327341.s001.tif]
