# Supplementary material for: pET28g: A Golden Gate-compatible pET vector for protein expression in Escherichia coli, validated by production of functional human ACE2
Source: PLoS One. 2025 Jul 7;20(7):e0327341. doi: 10.1371/journal.pone.0327341 (PMC12233280; doi:10.1371/journal.pone.0327341)
Supplement: S1 Table — (DOCX) [file pone.0327341.s002.docx]

**S1 Table - List of acceptor plasmids with predefined fusion sites available in the MoClo Toolkit (Addgene, kit # 1000000044)**

| Plasmid ID | 5' fusion site | 3' fusion site |
| --- | --- | --- |
| pAGM1276 | 1 | 2 |
| pICH41258 | 2 | 3 |
| pAGM1299 | 3 | 4 |
| pAGM1301 | 4 | 5 |
| pICH53388 | 5 | 6 |
| pICH53399 | 6 | 7 |
| pICH41264 | 3 | 5 |
| pICH41276 | 5 | 7 |
| pAGM1287 | 2 | 4 |
| pICH41308 | 2 | 5 |
