# Supplementary material for: pET28g: A Golden Gate-compatible pET vector for protein expression in Escherichia coli, validated by production of functional human ACE2
Source: PLoS One. 2025 Jul 7;20(7):e0327341. doi: 10.1371/journal.pone.0327341 (PMC12233280; doi:10.1371/journal.pone.0327341)
Supplement: S1 Protocol — The protocol is deposited under the https://doi.org/10.17504/protocols.io.j8nlk9e75v5r/v1 and can be accessed through the link: https://www.protocols.io/view/preparation-of-level-0-modules-for-golden-gate-ass-j8nlk9e75v5r/v1. (PDF) [file pone.0327341.s003.pdf]

Preparation of level 0 modules for Golden Gate assembly in pET28g, a new tool to prepare pET-based vectors for protein expression in *Escherichia coli*

RESERVED DOI:
10.17504/protocols.io.j8nlk9e75v5r/v1

Ines M. Luis[1], Isabel A. Abreu[1]
[1]ITQB NOVA

Isabel A. Abreu
ITQB NOVA

Protocol Info: Ines M. Luis, Isabel A. Abreu . Preparation of level 0 modules for Golden Gate assembly in pET28g, a new tool to prepare pET-based vectors for protein expression in Escherichia coli. **protocols.io** **https://protocols.io/view/preparation-of-level-0-modules-for-golden-gate-ass-drwh57b6**

Manuscript citation:
Luís IM, Parada M, Vicente JB, Abreu IA. pET28g: A Golden Gate-compatible pET vector for protein expression in *Escherichia coli*, validated by production of functional human ACE2. PLoS ONE. 2025 (to be published in association with this protocol).

Created: November 18, 2024

Last Modified: June 05, 2025

Protocol Integer ID: 112297

Keywords: pET28g, pET system, Golden Gate, Escherichia coli, Recombinant protein, Protein expression

Funders Acknowledgements:
Fundação para a Ciência e a Tecnologia (Portugal)
Grant ID: Fellowship for IML: PD/BD/113982/2015
Fundação para a Ciência e a Tecnologia (Portugal)
Grant ID: Green-it Base Funding: DOI 10.54499/UIDB/04551/2020
Fundação para a Ciência e a Tecnologia (Portugal)
Grant ID: Green-it Programatic Funding: DOI 10.54499/UIDP/04551/2020
Fundação para a Ciência e a Tecnologia (Portugal)
Grant ID: FilliGRAIN-PROTECT grant: DOI 10.54499/PTDC/ASP-PLA/1920/2021
Fundação para a Ciência e a Tecnologia (Portugal)
Grant ID: S4FUTURE Associated Laboratory: DOI 10.54499/LA/P/0087/2020

Abstract

The pET28g is a plasmid for protein expression in *Escherichia coli*. It was built from pET28a, where a Golden Gate cassette containing the *lacZα* reporter gene was cloned. The assembly of pET28g is based on Golden Gate cloning, and it was designed as an extension of the MoClo Toolkit (Kit #1000000044, Addgene). The pET28g plasmid and all the level 0 modules prepared in the lab are deposited at Addgene. This protocol provides guidelines to understand the pET28g system nomenclature and instructs through the preparation of level 0 modules and assembly of the final vector to express your construct of interest using the pET28g.

Guidelines

The pET28g is a plasmid for protein expression in *Escherichia coli*, built from pET28a.
The Golden Gate cassette was cloned in the pET28a, 6 nucleotides upstream of the Shine-Dalgarno sequence (ribosome-binding site, RBS), using the NcoI cleavage site available in the pET28a (see Figure 1 for more details). To establish pET28g as an extension for the MoClo Toolkit (Kit #1000000044, Addgene), the **pre-defined cloning sites were adapted and numbered to facilitate the nomenclature of level 0 modules** (see Table 1 for fusion sites numbering).

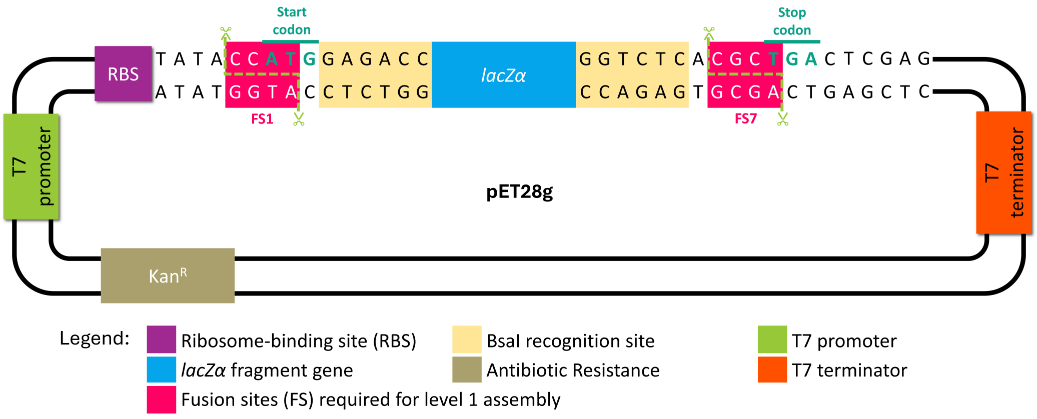

Figure 1 – Schematic representation of the pET28g plasmid with the elements of the Golden Gate cassette inserted depicted. The CCAT fusion site was chosen because it complements the NcoI and allows the reconstitution of the ATG site in exactly 6 base pairs upstream of the RBS. Adapted from Luís et al. (2025) [1].

A	B	C	D	E	F	G	H
Fusion sites that can be assembled in tandem (5' to 3')	CCAT	AATG	AGGT	TTCG	GCTT	GGTA	CGCT
Fusion site (FS) identification	1	2	3	4	5	6	7

Table 1 – Identification of the pre-defined fusion sites

Following the IDs attributed in Table 1, the **level 0 nomenclature should follow this structured nomenclature: *p028g##_ELEMENT.*** *p028g* identifies the vector as a level 0 vector for the pET28g system; *##* identifies the fusion sites that flank the element cloned in the vector; *ELEMENT* must identify the element cloned in the vector.

Safety warnings

Do not forget to plan level 0 modules so the tags and protein of interest are kept in the same reading frame.
For successful cloning using Golde Gate technology, it is fundamental that all the DNA inserted in the level 0 modules contains no recognition sites for the type IIS restriction enzymes used for construct assembly (BsaI and BpiI). When planning the construction of level 0 modules, remove all the type IIS recognition sites using synonymous point mutations to maintain the final amino acid sequence.

Before start

Level 0 modules must be prepared to be assembled in tandem in the desired order and complete the transcription unit in pET28g. Level 0 modules can be coding sequences; any desired tags at N- and/or C-terminal to facilitate protein purification, solubilization, or detection with antibodies; protease cleavage sites; and signal peptides. As in any Golden Gate assembly, the DNA sequences to prepare level 0 modules must be domesticated, and the **level 1 assembly must be planned so that all level 0 modules assemble in tandem with matching fusion sites.** Don't forget to **add any necessary nucleotides to ensure the whole coding sequence is kept in the same reading frame.**

Level 0 modules are prepared by assembling the desired element in existing plasmids. Two types of acceptor plasmids can be used, depending on the fusion sites to be added to the level 0 modules in preparations. The MoClo kit contains several level 0 acceptor plasmids with pre-defined fusion sites and a universal acceptor plasmid for when the desired fusion sites combination is not available. Table 2 below summarizes the plasmids with pET28g fusion sites available in the MoClo Toolkit; for a comprehensive list of MoClo plasmids with pre-defined fusion sites, see Engler et al. (2014) [2]. Depending on the plasmid used to prepare the level 0 module, different adaptors must be added to the DNA fragment of interest (FOI). Plus, depending on the size of the FOI and the availability of the DNA for amplifications, 3 strategies can be pursued to get the FOI ready for cloning: 1) FOI may be synthesized with the proper adaptors; 2) FOI may be amplified from a template using primers with the proper adaptors; and 3) oligonucleotides may be annealed to generate elements with less than 100 bp. Figures 2 and 3 depict these 3 different strategies and the adaptors necessary for the two types of acceptor plasmids. See Luís et al. (2025) [1] for detailed explanations on these different cloning strategies.

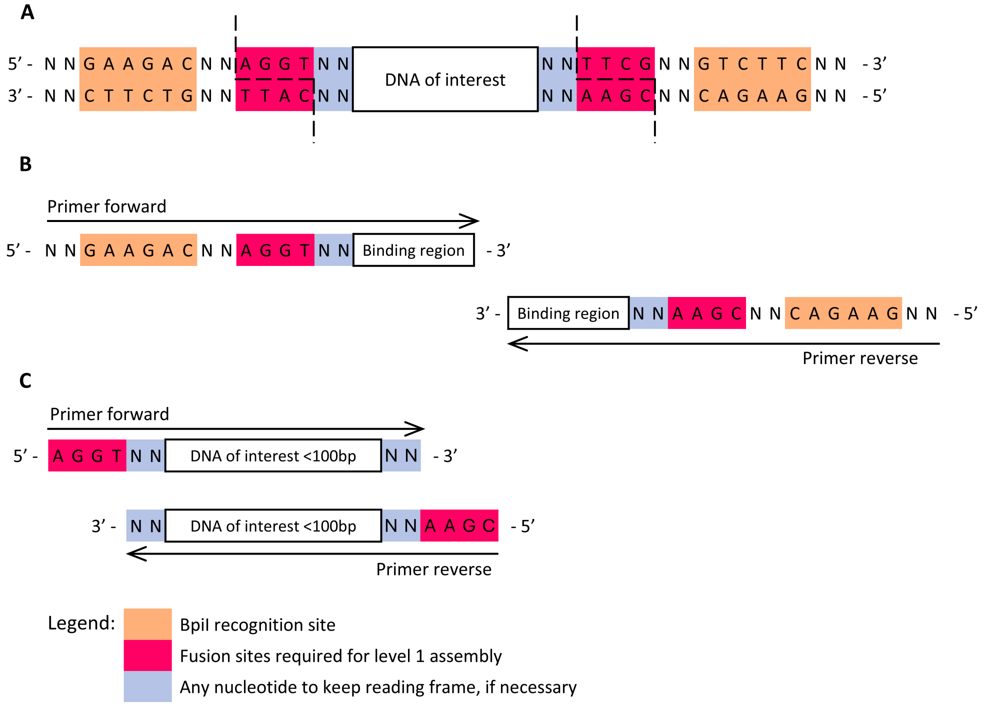

Figure 2 - Schematic representations of different approaches to preparing the FOI for cloning level 0 modules using a plasmid with the pre-defined fusion sites. The plasmid pAGM1299 with fusion sites 3 (AGGT) and 4 (TTCG) is used as an example in this schematic representation. (A) Synthesis of the DNA of interest including the BpiI recognition site and fusion sites as depicted in the figure; (B) Amplification of the DNA of interest using primers with adaptors that contain the BpiI recognition site and fusion sites as depicted in the figure; and (C) Synthesis of oligonucleotides with complementary sequences in the region of the DNA of interest and uncomplemented fusion sites in the overhangs as depicted in the figure. In this last strategy, no type IIS enzyme is used to expose the fusion sites, as the overhangs are ready to ligate upon oligonucleotide annealing. Adapted from Luís et al. (2025) [1].

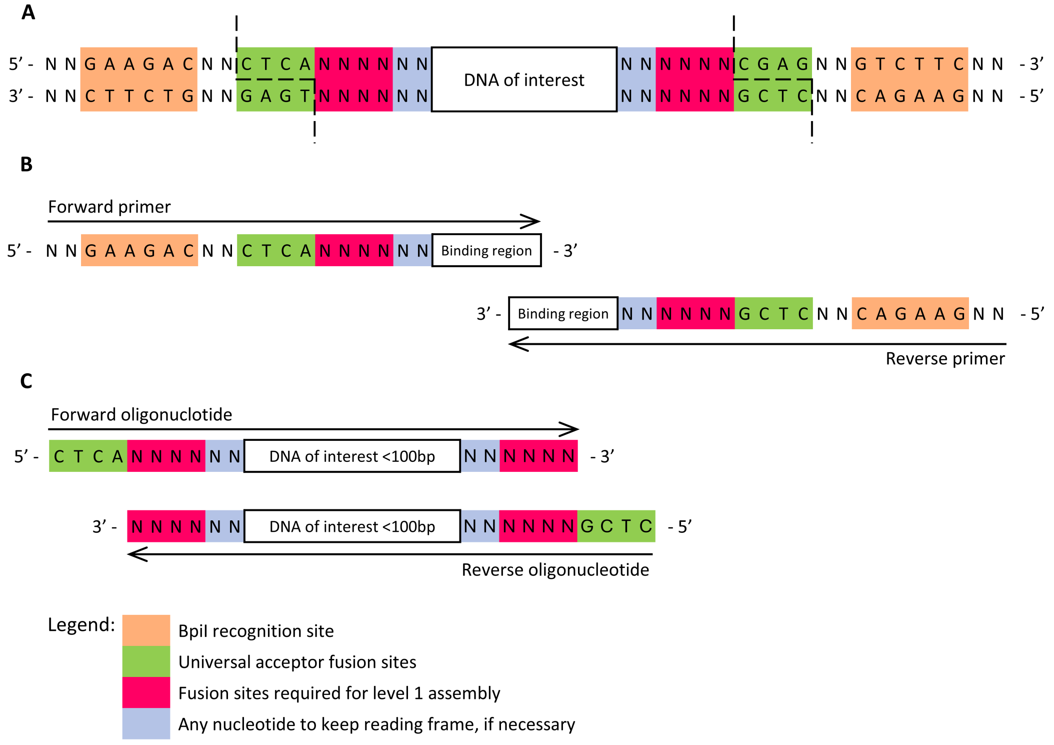

Figure 3 - Schematic representations of different approaches to preparing the FOI for cloning level 0 modules using the universal acceptor plasmid (pAGM9121). (A) Synthesis of the DNA of interest including the BpiI recognition site, the universal acceptor fusion sites, and the fusion sites for level 1 assembly as depicted in the figure; (B) Amplification of the DNA of interest using primers with adaptors that contain the BpiI recognition site, the universal acceptor fusion sites, and the fusion sites for level 1 assembly as depicted in the figure; and (C) Synthesis of oligonucleotides with complementary sequences in the region of the DNA of interest and fusion sites for level 1 assembly and uncomplemented fusion sites in the overhangs as depicted in the figure. In this last strategy, no type IIS enzyme is used to expose the fusion sites, as the overhangs are ready to ligate upon oligonucleotide annealing. Adapted from Luís et al. (2025) [1].

MoClo Toolkit plasmid ID	5' fusion site	3' fusion site
pAGM1276	1	2
pICH41258	2	3
pAGM1299	3	4
pAGM1301	4	5
pICH53388	5	6
pICH53399	6	7
pICH41264	3	5
pICH41276	5	7
pAGM1287	2	4
pICH41308	2	5

Table 2 - List of acceptor plasmids with predefined fusion sites available in the MoClo Toolkit (Addgene, kit # 1000000044)

When using an acceptor plasmid with pre-defined fusion sites (Table 2), the fusion sites to ligate the FOI with the acceptor plasmid are also the fusion sites used to assemble the level 1 construct. The FOI must be

prepared to expose the fusion sites upon digestion with the BpiI enzyme (Figure 2). Recognition sites for the BsaI enzyme (GGTCTC) exist in the cloned acceptor plasmid and will expose the fusion sites during level 1 assembly.

When using a universal acceptor plasmid, the fusion sites to ligate FOI with the acceptor plasmid are unique and different from the fusion sites used to assemble the level 1 construct. FOI must be prepared to, upon digestion with the BpiI enzyme, expose the sequences CTCA and CGAG in the 5' and 3', respectively (Figure 3). These sequences must then be followed by the fusion sites used to assemble the parts in level 1. CTCA and CGAG sequences will reconstitute BsaI recognition sites (GGTCTC) in the cloned acceptor plasmid and will be used to expose the fusion sites during level 1 assembly (see Luís et al. (2025) [1] for more detailed explanations).

Prepare the insert of the DNA fragment of interest (FOI) for level 0 assembly

1 **Option 1 – Synthesize FOI**

1.1 Design your FOI with the proper adaptors (see Figures 2A and 3A)

1.2 Order DNA synthesis of your FOI

2 **Option 2 -Amplify FOI from a template**

2.1 Design primers to amplify your FOI with the proper adaptors (see Figures 2B and 3B)

2.2 Amplify FOI with a proofreading DNA polymerase

2.3 Purify the PCR product from solution or gel

3 **Option 3 - Anneal oligonucleotides to prepare small DNA element**

3.1 Design oligonucleotides complementary in the FOI region with the proper overhangs (see Figures 2C and 3C)

3.2 Resuspend oligonucleotides to a final concentration of 100 µM with MilliQ H_2O or TE buffer.

3.3 Set up the annealing mixture:

	Volume	Reagent
	46 µL	Annealing Buffer (10 mM Tris pH 8.0, 1 mM EDTA, 50 mM NaCl)
	2 µL	Forward Oligonucleotide (100 µM)
	2 µL	Reverse Oligonucleotide (100 µM)

3.4 Anneal oligonucleotides using the following program:

Step	Temperature	Time and cycles
Step 1	95 °C	5 min
Step 2	95 °C	1 min and decrease 1 °C per cycle until reach annealing temperatures
Step 3	Annealing temperature	30 min
Step 4	Annealing temperature	1 min and decrease 1 °C per cycle until reach 25 °C
Step 5	8 °C	pause

An example of a pair of oligonucleotides to anneal with an annealing temperature of 52 °C:

Step	Temperature	Time	Go to step	No. of cycles	Options
Step 1	95 °C	5 min			
Step 2	95 °C	1 min	2	42*	-1 °C per cycle
Step 3	52 °C	30 min			
Step 4	52 °C	1 min	4	26**	-1 °C per cycle
Step 5	8 °C	pause			

*95°C - 52°C = 43, then 42 cycles are necessary to decrease the temperature from 95 °C to 52°C
**52°C - 25°C = 27, then 26 cycles are necessary to decrease the temperature from 52 °C to 25°C

Assemble the level 0 plasmid

4 Set up the mixture to ligate the insert and the level 0 acceptor

Amount	Reagent
100 ng	Level 0 acceptor plasmid
300 ng	DNA insert prepared before
2 µL	T4 ligase buffer
1 µL	T4 ligase
0,5 µL	BpiI
Up to 20 µL with H2O	

5 Digest and ligate using the following program:

Step	Temperature	Time	Go to step	No. of cycles
1. Digestion	37 °C	20 min		
2. Ligation	16 °C	10 min	1	2
3. Final Digestion	37 °C	20 min		

Step	Temperature	Time	Go to step	No. of cycles
4. Enzyme inactivation	65 °C	20 min		
	8 °C	pause		

6 Transform 100 µL of *E. coli* DH5α competent cells with 5 µL of the ligated mixture

7 After 1 hour of recovery, plate 10 to 20 % of the DH5α transformed cells in LB plates containing 100 mg/L spectinomycin, 100 mg/L X-Gal, and 100 µM IPTG.

8 Incubate plates overnight at 37°C.

9 Streak white CFUs from the LB plate and use one of these cultures to extract the plasmid.

10 Digest extracted plasmid with restriction enzymes to validate it.

11 If necessary, sequence the plasmid to ensure that the DNA of interest has no mutations.

Assemble pET28g (level 1 plasmid)

12 Set up the mixture to ligate the insert and the level 0 acceptor

Amount	Reagent
100 ng	pET28g
300 ng	each level 0 module
2 µL	T4 ligase buffer
1 µL	T4 ligase
0,5 µL	BsaI
Up to 20 µL with H_2O	

13 Digest and ligate using the following program:

Step	Temperature	Time	Go to step	No. of cycles
1. Digestion	37 °C	20 min		
2. Ligation	16 °C	10 min	1	2
3. Final Digestion	37 °C	20 min		
4. Enzyme inactivation	65 °C	20 min		
	8 °C	pause		

14 Transform 100 µL of *E. coli* DH5α competent cells with 5 µL of the ligated mixture.

15 After 1 hour of recovery, plate 10 to 20 % of the DH5α transformed cells in LB plates containing 100 mg/L kanamycin, 100 mg/L X-Gal, and 100 µM IPTG.

16 Incubate plates overnight at 37°C.

17 Streak white CFUs from the LB plate and use one of these cultures to extract the plasmid.

18 Digest the extracted plasmid with restriction enzymes to validate it.

Protocol references

[1] Luís IM, Parada M, Vicente JB, Abreu IA. pET28g: A Golden Gate-compatible pET vector for protein expression in *Escherichia coli*, validated by production of functional human ACE2. PLoS ONE. 2025 (to be published in association with this protocol).
[2] Engler C, Youles M, Gruetzner R, Ehnert TM, Werner S, Jones JDG, et al. A Golden Gate modular cloning toolbox for plants. ACS Synth Biol. 2014;3: 839–843.

Acknowledgements

The authors acknowledge funding by FCT - Fundação para a Ciência e a Tecnologia, as detailed in the section "Funders Acknowledgement".
